# Annual Address

**Published:** 1872-05

**Authors:** Geo. M. McDowell

**Affiliations:** President, Barnesville, Georgia


### ANNUAL ADDRESS,

*Delivered Before the Georgia Medical Association, Twenty-Third Session, Held in Columbus, Georgia, April* 10, 1872, *by the President,*

GEO. M. McDOWELL, M.D., OF BARNESVILLE, GEORGIA.

*Gentlemen of the Association,*—Custom and the requirements of your by-laws both demand of your presiding officer that he shall precede the strictly official duties of the station with an address, in which there shall be found an expression of good-will, if nothing more substantial, for your consideration. In compliance with this duty, I would be untrue to the sentiments of my heart were I to fail to impress my fellow-members with the grateful feelings entertained toward them, and with the absorbing interest I feel in the great objects for which our Association was formed.

In addressing an audience of intelligent gentlemen, trained to habits of reading and reflection on every subject in the vast domain of literature and science, it is no easy task to select a theme which may be invested with sufficient interest to claim attention, and sufficient novelty not to be irksome.

These annual gatherings of our profession bring together its membership in pleasant reunion, and afford an opportunity for relaxation from the cares and toils of business, besides furnishing a convenient occasion for taking a survey of the interests and duties of the Association—to determine what of good it has accomplished in the past, and what may remain to engage its attention and energies in the future. They are mile-stones on the road of professional progress, where we may pause a moment to cast a backward glance over the road we have traveled, and estimate the distance yet to be gained, and the labors of the journey.

In reviewing the past, the inquiry readily suggests itself—Whether the Association has accomplished wholly, or in part, the objects of its organization? In seeking for an answer to this question, we look not alone to the achievements of our own body, but also to results of the labors of other similar ones, in conjunction with the American Medical Association—all working, in their respective spheres, for the accomplishment of the same great ends: the advancement of medical science, the improvement of the profession itself, and increase of its efficiency, and the promotion of public good. A very superficial glance at the state of the medical mind of America prior to the formation of the National Association, about twenty-five years ago—its very general stagnation, the absence of high and noble aspirations, the tendency to predominance of material interests in both the educational and practical departments—and a contrast of these with the spirit of improvement that now pervades the mass of medical society, the earnest efforts for self-culture and promotion of the public welfare, will readily convince us that the profession has been steadily advancing in its onward and upward career.

It is not too much to assert that these grand results could never have been attained by the individual members in their isolation. The annual deliberations of the earnest men of the profession in council, the practical inquiry originated, with their various legislation, have been the fruitful means of arousing a general and determined spirit of progress; while in bringing together from their quiet retirement many who would otherwise never meet, leading to an interchange of thought and sentiment in the social assemblage and around the festal board, our Association has destroyed the excessive self-respect and broken down the narrow and selfish feelings which are the effect of isolation, and greatly developed the better qualities of our nature. Now and then, perhaps, disturbing elements of a foreign nature may cause an apparent halting, but they can not long retard the irresistible force of the moving mass of the profession.

The first of the great objects of our associations was the improvement of the profession itself, and an essential element of success in this has been a higher standard of qualification in those who seek to enter its ranks. So keenly has this necessity been felt, and so generally recognized, that it has been the theme of three-fourths of the addresses before the annual meetings of the State and National Associations, the subject of perpetual reports of committees and individuals, and of resolves and recommendations to colleges and private preceptors—all urging the requirement of improved preparatory education, longer terms of professional study, a junction of clinical and didactic instruction, and a more thorough examination before admission to the honor of the doctorate. Possessing no power to exercise coercive measures, and relying alone upon appeals to the judgment and conscience of those concerned, it is not a matter of surprise that ALL of the high aims and reasonable wishes have not been realized. Indeed, there are some who assert that *no advance* has been made in this respect, and who are ready to submit, in sadness and despair, to evils which they conceive to be without remedy.

Those who hailed the grand awakening of the profession throughout the land, that attended the formation of these organizations, as the approach of a "professional millenium," and who expected the instant and miraculous disappearance of all those noxious influences that were sinking the profession to a mere business, have been disappointed; and although it is a lamentable fact, that, as regards medical education, the efforts have been *greater*, and the *results less*, than in any other field of labor, yet a candid investigation of facts will satisfy the honest enquirer that these efforts have not been barren of fruit—that a higher standard of preliminary education than formerly existed has generally characterized young men entering the profession within the last twelve or fifteen years.

Although much remains to be done, so far from being discouraged by partial failure, and abandoning the object as an unattainable good, it is our duty to reiterate, year after year, our convictions, wishes, and *demands*, until complete and permanent reform shall have been effected, and our noble calling thoroughly purified and regenerated. Then, instead of "insensible shades," we shall have broad and distinct lines of demarkation between quackery and regular medicine.

If any plan can be suggested, State or National, for applying to graduates of all the schools the same test of merit and qualification, before conferring the degree, or license to practice, it will constitnte a long stride on the march of improvement. In the present state of social and professional advancement, there is not the least excuse for admitting to membership any whose preparatory education is not up to the highest possible standard. There is no dearth of doctors, in any part of the wide world, that demands a continuance of the hot-bed system that has characterized American schools.

Besides, the range of proper professional study is so extensive as to require the whole energies of an active mind, not only during a college term, but throughout a long life-time, allowing no time to supply primary deficiencies after entering the profession, without falling behind in the race. I feel quite sure that no class of our brethren will more fully indorse this assertion than those honest and earnest workers among us who themselves have had to perform the double task of repairing early discrepancies, while struggling to keep abreast of the onward march of science.

A glorious epoch in the history of the efforts of the profession for self-improvement was that which gave us the "Code of Medical Ethics." Like all human productions, it is not perfect; but, instead of creating discord in its enforcements, as has been feared in high places of late years, I am fully persuaded that a sincere observance of its rules would maintain harmony in the profession, and effectually prevent those discreditable feuds, arising from petty jealousies and imaginary opposition of interests, which so greatly damage the profession in the public esteem. One of the most distinguished of American physicians and teachers once said: "I do honestly believe that, to a young physician going forth into a life full of moral conflicts, the wearing of this ægis would be one of his surest defenses; the next to the Holy Scripture and the grace of God, it would serve most effectually to guard him from evil." Let us reflect whether it is not well to recur to these "first principles."

With regard to another great aim of our own and similar Associations, the advancement of medical science and improvement of medical literature, their labors have been successful to a most gratifying extent. Thirty years ago, American publishers seldom issued any but reprints of foreign works; while now, books of a high grade of literary and scientific merit, by American authors, may be procured by the enquirer in any department of medicine. This result is due in a great measure to appeals made from time to time by the State Associations, but especially by the National Association, to native talent and tional pride. The great impetus given to medical journalism, meanwhile, and which may have been carried a trifle too far, may be traced to the same influences.

Besides all these, we have the immense volumes of "Transactions," which are the repositories of most valuable information. Like all the large books that were ever written, they contain a great deal of superfluous and chaffy matter; but many of the best intellects of the nation have contributed to their solid contents, while in many cases the choicest productions of the best American authors, and the most brilliant achievements of our host of scientific investigators, have made their first appearance to the world through this medium.

A commendable measure for stimulating professional labor and scientific research, by our own Association, was that of awarding prizes for papers of unusual merit. This, I regret to say, has lapsed into disuse for several years—the last prize having been awarded by your committee to a learned physician of another State, in 1867. How shall we account for this? Are we becoming indolent? Shall we fall behind our brethren of other States in cultivating the inviting fields of science, and extending the boundaries of knowledge?

In our efforts for the promotion of the general good of the community, much, very much, has been accomplished; but much more remains to invite your earnest labors. In the department which has properly been denominated "State Medicine," the harvest is ripe and inviting. It is in this direction that your influence may be most appropriately exerted, and your greatest triumphs be realized.

It is just here the potency and beneficence of our efforts, as an *organized body,* may be most effectually exemplified. Individual labor is equal to the task of making the deepest research into physiology, pathology, chemistry, etc., and of ranging its trophies in their proper order in the great store-house of science. But the great interests of the community, relating to the influences that affect their health—such as quarantine, sanitary police, vaccination, registration, etc.—can only be controlled by the voice of the profession as a body, expressed to the legislative and municipal councils of the country. Far greater advances have been made in the prevention than in the cure of disease during the present century, and yet, in some large cities of the most enlightened nations, the mortality is not less than it was a hundred years ago. It is greater in all than it would be, were municipalities to adopt systematic sanitary regulations in accordance with the present state of medical knowledge. The collection and preservation of vital statistics—registration of deaths, births, etc.—the study of climatology and epidemics—the preservation of the health of cities—the adoption of measures to prevent the spread of the insidious teachings and practices, so prevalent in some sections, which tend to decrease population and sap the moral foundation of society—these should be, henceforth, the subjects of your earnest consideration, as they are of vital interest alike to the physician and statesman.

To render your action effective in enlightening the public mind and procuring legislative enactment, it would be in the highest degree beneficial to secure the organization of a State Board of Health, such as have lately been organized by law in one or two of the States. The fearful manner in which small-pox has been rioting during the last winter, in many of the Northern cities, admonishes us that the profession has not discharged its duty in warning the community of the danger, or else the authorities have been guilty of criminal negligence in allowing food for the pestilence to accumulate, in the large number of unprotected persons. It hardly admits of question, that some system of compulsory vaccination ought to be established. So soon as the distrust growing out of the discussion as to the transmission of other constitutional diseases with the vaccine virus, shall have died away, there will probably be no difficulty in effecting this very necessary legislation.

Your body, at the last session, took a step with regard to the establishment of an inebriate asylum, which seems to be justified by the enlightened judgment of the profession and the civilization of the age. Intemperance is now considered by many intelligent physicians as a positive disease that should be subjected to treatment, and that may be cured "in the same sense that other diseases are." The "American Association for the Cure of Inebriates," organized last year in New-York city, and composed of physicians connected with inebriate asylums in different States, declares that—"All methods heretofore employed having proved insufficient for the cure of inebriates, the establishment of asylums for such a purpose is the great demand of the age;" and that "Every large city should have its local or temporary home for inebriates, and every State, one or more asylums for the treatment and cure of such persons."

Whether inebriety be regarded as a disease or not, it can not be denied, that any plan which will restore the unfortunate subjects of intemperance to sobriety will be a boon to thousands of wretched families, and is worthy the attention of the physician and the philanthropist.

The charge—so prominently and offensively made in certain quarters by the more ultra and fanatical portion of those engaged in the good work of temperance reform, and to which some *super-compunctious* medical men, here and in England, have "confessed judgment"—that physicians are "largely responsible" for the vast amount of drunkenness, and its attendant evils, on account of prescribing alcoholic tinctures, is, in my opinion, almost without the shadow of foundation in fact. Whatever else may be urged against that form of administration, it is quite certain the habit of drunkenness can not, in any appreciable number of cases, be fairly attributed to it, as I believe nineteen-twentieths of this professional audience would readily verify. Nevertheless, the evils of intemperance are so widespread and so manifest, and its effects on the innocent and helpless offspring of the inebriate so shockingly abhorrent to every lover of his race, that we, in common with all classes of our fellow-citizens, should be ready to join in any measures likely to result in its abridgment. It is a question for serious consideration, whether you ought not to exert your influence with the Legislature for prohibitory enactments.

Let us contemplate for a moment a theme less practical in its nature, but not less disagreeable to the sincere lover of truth and the true medical philosopher. I refer to the obvious tendency to religious infidelity and *atheism* in the teachings of many who hold high and influential places in the profession.

Every careful student of histology has been intensely interested in the researches of old and modern laborers in that branch of science, the "fibre theory" of Haller, the "globular" hypothesis, and Brown's "nucleus," and thrilled with the steadily increasing brilliancy of the revelations of the microscope which have attended the evolutions of the great "cell doctrine." But whilst deriving so much pleasure and instruction from the labors in *ultimate* physical analysis—from Huxley, Bennett, Beale, Virchow, Tyson, and the rest—we have been repeatedly shocked by the materialism and *atheism* constantly cropping out from the writings of some of these authors, in their "side glances" at the phenomena of consciousness and thought. Several of these "physicists" find no difficulty in attributing all the phenomena of life to physical causes alone—to chemical actions and reactions—and denying the existence of a vital principle, exerting its "mysterious sustaining and controlling power" in every microscopic cell, every nerve fibre and muscular fibril in the living organism. So long as their infidel doctrines were left to inference, or modestly avowed, it was not believed that the professional mind was in any great danger, or that this "era of infidelity" would exist longer than the time required to manifest its fallacy. But there is cause for alarm when the most renowned medical teacher of the age—one whose doctrines in the development of science receives the unquestioning confidence of the profession of *two continents*—boldly avows these atheistic views, and declares open war on religion and those deluded and ignorant people who believe in God and spirits. Such were the declarations of Virchow, at Rostock, in Germany, a few weeks ago—made, as he asserted, in the name and for the interest of science! And it is said they were received with great applause by a vast audience of scientists, largely composed of medical men.

Leaping from the domain of facts to that of ideas, from physics to metaphysics, the scientific giant becomes the theological pigmy, and he whose hard-earned facts and beautiful theories constitute an important chapter in the great book of science, presents himself to our astounded view as the intolerant advocate of the grossest materialism! Having abolished God and all spiritual existence, he stands appalled at the result, and essays to allay the consternation of the moral world by "hoping that a freer knowledge of science will awaken higher moral aims," and thus the incentives to virtue will be perpetually increased!

This school of atheists, having satisfactorily evolved "life," whether of plant or animal, from the mere operations of physical causes, still leave unaccounted for certain *forces* which they recognize in nature, and which would exist were there no material for the forces to act on, together with matter itself—"the ultimate physical element." Whence do *these* come? Do they spring spontaneously into existence? Or were they created by the Divine Author as *agents* to be employed by the *vital power* to accomplish the functions of life? Busy with his physical science, and infatuated with his new worship, this great scientist furnishes an illustrious exemplification of

——"him that vexed his brain and theories built
Of gossamer upon the brittle winds;
Perplexed exceedingly why shells were found
On mountain-tops: but wondering not
Why shells were found at all, more wondrous still."

Though these infidel absurdities in high places mostly manifest themselves in foreign lands, it will be difficult to avert the transplanting of them to our own shores, along with other instructions from the same source. It is your duty to combat them, since they have assumed the aggressive. It is equally your duty to aid the lovers of science and literature in every walk of life—to rescue the national character from the gross commercial philosophy that subjects every proposition to the test of utilitarianism, dwarfs the intellect, and clips the wings of genius.

To cultivate successfully the field of labor before you, you must have a working membership. The ranks of the medical profession furnish no place for the indolent and slothful man. To acquire a knowledge of the vast store of facts and principles contributed by their predecessors and contemporaries—to contribute something themselves to the same common fund—to become well-versed in the literature of the profession, and give sufficient attention to general literature—will require great industry. The apology which I offer for pressing these reflections on your attention is, that I am convinced that the chief obstacle to professional advancement is the laziness of its members. There is too general a disposition to consider the task ended when the doctorate is attained, as if the brief college term and the student's text-books supplied the sum of professional knowledge, or at least all that could be deemed necessary beyond that to be reaped in the vaunted "experience" of the individual. There is abundant reason to believe, that of all the hindrances to improvement in the profession, not even excepting the imperfect primary education, this want of habits of study, among physicians in active practice, is the greatest.

One of our most careful and accurate observers*, who is in a position to know the facts, says:—"Throwing aside those books which have to be bought by students as text-books, and those others which physicians must have for constant reference—dictionaries, dispensatories, and the like—we find that the wants of the fifty-odd thousand doctors in the United States are supplied abundantly by an average edition of a thousand, or at most fifteen hundred, copies; and the book-seller is fortunate if he does not find many of these hanging heavily on his hand for months and years."

Making due allowance for the distrust of novelties, often amounting to obdurate prejudice, I have been amazed at the large number of intelligent physicians who deny themselves and their patients the benefit of two of the most valuable acquisitions in diagnosis and therapeutics, not now very recent—the body thermometer, and that little *talisman* that so often brings magic relief to both physician and patient, the hypodermic syringe.

We have, however, quite an army of indefatigable workers and students, whose active brains and dexterous hands are illu-

*Editor of the *Medical and Surgical Reporter*, Philadelphia.

minating the pathway and facilitating the progress of the lover of learning, whose unremitting, arduous labor and self-imposed tasks would be appalling to the sluggards, to be seen far in the rear, dragging their heavy footsteps along. May we not hope we are approaching an era when the whole lump is to be leavened. No man can be happy without exercising the virtue of a cheerful industry.

> "Self-ease is pain; thy only rest
> Is labor for a worthy end—
> A toil that gains with what it yields,
> And scatters to its own increase;
> And hears, while sowing outward fields,
> The harvest-song of inward peace."

